# Prenatal Ethanol Exposure Leads to Attention Deficits in Both Male and Female Rats

**DOI:** 10.3389/fnins.2020.00012

**Published:** 2020-01-24

**Authors:** Ruixiang Wang, Connor D. Martin, Anna L. Lei, Kathryn A. Hausknecht, Keita Ishiwari, Jerry B. Richards, Samir Haj-Dahmane, Roh-Yu Shen

**Affiliations:** ^1^Department of Pharmacology and Toxicology, Jacobs School of Medicine and Biomedical Sciences, University at Buffalo, The State University of New York, Buffalo, NY, United States; ^2^Department of Psychology, University at Buffalo, The State University of New York, Buffalo, NY, United States

**Keywords:** fetal alcohol spectrum disorders, attention-deficit/hyperactivity disorder, impulsivity, lapse of attention, prenatal ethanol exposure

## Abstract

**Background:**

Prenatal ethanol exposure (PE) causes multiple behavioral and cognitive deficits, collectively referred to as fetal alcohol spectrum disorders (FASD). Studies show that 49–94% of FASD children exhibit attention deficits, even when they have normal IQs or lack severe facial deformities, suggesting that attention deficits could be caused by even moderate prenatal exposure to alcohol, of which the underlying neural mechanisms are still unclear. A valid rodent model could help elucidate this phenomenon.

**Materials and Methods:**

A second-trimester equivalent binge drinking PE model was utilized. Pregnant Sprague Dawley rats were administered with 15% (w/v) ethanol (6 g/kg/day, via gastric gavage) during gestational days 8–20, and their offspring were the subjects in the present study. A modified 2-choice reaction time (2-CRT) task was used to illustrate possible attention deficits, including increased action impulsivity and lapses of attention. Enhanced impulsivity was reflected by more premature responses while increased lapses of attention were manifested as more incorrect responses and/or greater variability of reaction time, demonstrated by more skewed distributions of reaction time. Ten-week-old male and female rats were tested for three sessions following 16–19 days of training.

**Results:**

Our PE paradigm caused no major teratogenic effects. PE led to increased impulsivity exhibited as greater premature responses and augmented lapses of attention shown by greater skewnesses of reaction time distributions, relative to controls. The deficits were observed in both PE male and female rats. Interestingly, in males, the attention deficits were detected only when the 2-CRT task was relatively difficult whereas in females they were detected even when the task was at a less demanding level.

**Conclusion:**

We show that the binge drinking pattern of PE led to attention deficits in both sexes of rats even though no major teratogenic effects were observed. Therefore, this rodent model can be used to study neural mechanisms underlying attention deficits caused by PE and to explore effective intervention approaches for FASD.

## Introduction

Prenatal ethanol exposure (PE) could lead to physical, behavioral, and cognitive deficits, collectively referred to as fetal alcohol spectrum disorders (FASD). Estimates indicate that the global prevalence of FASD is 0.8% in the general pediatric population (Lange et al., 2017) whereas it is as high as 2–5% in the United States (May et al., 2009, 2014). Such high prevalence suggests that it is imperative to understand the mechanisms underlying PE-induced impairments and design effective intervention strategies.

One of the major conditions caused by PE is attention deficits. Attention-deficit/hyperactivity disorder (ADHD) is one of the most commonly occurring developmental disorders, with a prevalence rate of 5% in children and 2.5% in adults (American Psychiatric Association [APA], 2013). This disorder causes impairments in learning, loss of productivity, and huge costs of treatment and extra care. The incidence of ADHD in individuals with FASD is much higher than that in the general pediatric population (49–94 vs. 5%) (Bhatara et al., 2006; Fryer et al., 2007; Kingdon et al., 2016). Attention deficits are observed in children with FASD, even when they have normal IQs (Astley, 2010; Astley and Grant, 2012) or don’t show severe facial deformities (Kingdon et al., 2016) caused by heavy ethanol exposure. This observation suggests that even moderate PE could cause attention deficits. Another interesting observation is that ADHD and FASD patients differ qualitatively in cognitive impairments, including in attention deficits. Moreover, ADHD patients with and without FASD often respond differently to pharmacological treatments (Nanson and Hiscock, 1990; Coles et al., 1997; O’Malley et al., 2000; O’Malley and Nanson, 2002; Kingdon et al., 2016). Accordingly, it is tempting to speculate that there may be differences in neural mechanisms underlying attention deficits between FASD and other ADHD patients. As such, it is necessary to establish a valid rodent model to study attention issues specifically caused by PE.

**FIGURE 1:**
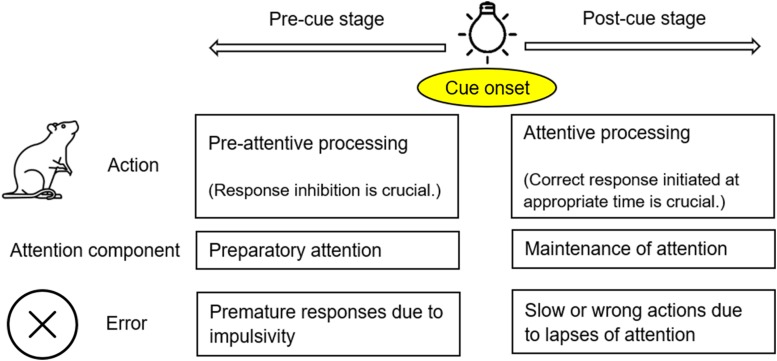
The theoretic framework of an attentional process applied in the present study. An attentional process can be divided into two stages: pre-cue and post-cue stages, separated by the onset of a cue/stimulus (e.g., the onset of light illumination). At the pre-cue stage, animals need to avoid being distracted; impulsivity (impaired response inhibition) may lead to premature responses. At the post-cue stage, animals need to initiate their responses at the appropriate time; lapses of attention may lead to slow or wrong actions.

Attention is a focalized, concentrated, and conscious cognitive process (James, 1890). It has been established that when animals pay attention to certain stimuli, they make efforts to process the relevant information while ignoring other targets (Watson and Breedlove, 2016). The attentional process can be divided into two stages: pre-cue and post-cue (Figure 1). At the pre-cue stage (before the target displays), animals need to hold their attention and avoid being distracted. This effort has been referred to as preparatory attention (LaBerge et al., 2000), or pre-attentive processing (Atienza et al., 2001). Attention deficits at this stage are mainly manifested as premature responding due to failed response inhibition/suppression (Swann et al., 2013). At the post-cue stage (after the target displays), animals need to maintain/sustain their attention while quickly moving toward their expected goals. This effort could be called maintenance of attention (LaBerge et al., 2000), or attentive processing, as opposed to pre-attentive processing at the previous stage (Theeuwes, 2010). In order to make a correct response to an external stimulus at the appropriate time, animals have to reach a balance between response inhibition (at the pre-cue stage) and response initiation/facilitation (at the post-cue stage) (Kok, 1999; Hardung et al., 2017). This two-stage theoretical framework (Figure 1) is in line with the way in which ADHD is characterized. In *the Diagnostic and Statistical Manual of Mental Disorders*, the 5th edition (DSM-5) inattention and hyperactivity-impulsivity are grouped together as one disorder – ADHD (American Psychiatric Association [APA], 2013). In fact, inattention and hyperactivity-impulsivity are manifested differently, but the two symptoms could be observed in the same individuals. Hyperactivity-impulsivity disrupts the pre-cue stage of attention, often evidenced by premature responses (Dalley et al., 2011). In contrast, inattention mainly impacts the post-cue stage, to the detriment of the task performance. Inattention could be very brief, thus referred to as a momentary lapse of attention in the literature (Weissman et al., 2006).

Impulsivity is a multifaceted construct (Evenden, 1999), which, in essence, is the tendency of acting out without adequate forethought or needed information due to a lack of self-control (Bari and Robbins, 2013). This propensity is observed in multiple mental disorders (Evenden, 1999). One type of impulsivity, action impulsivity, or disturbed response inhibition (Bari and Robbins, 2013), disrupts attentional processes. It has been proposed that the fundamental problem underlying ADHD is impaired behavioral inhibition (Barkley, 1997).

The momentary lapse of attention is another disrupting factor, causing consequences such as slow responses to stimuli and initiations of wrong moves (Castellanos et al., 2005; Weissman et al., 2006). Taken together, as mentioned earlier, attentional processes require a fine balance between behavioral inhibition and initiation, which leads to appropriately timed actions (Hardung et al., 2017). Impaired inhibition and initiation manifest themselves differently. As such, both need to be assessed in our animal model of attention deficits.

A variety of behavioral tasks have been developed to examine attention deficits in rodents (Bushnell and Strupp, 2009; Bari, 2015). One of the most widely used paradigms is the 5-choice serial reaction time task (5-CSRTT) (Bushnell, 1998; Robbins, 2002). This task, however, has a relatively complex procedure and involves multiple spatial components. It, therefore, may entail a high attrition rate, a spatial bias toward certain signals, and/or a protracted training process (Puumala et al., 1996; Passetti et al., 2002; Bari et al., 2008), which could introduce certain confounding factors, such as age, learning effects, and “enrichment” resulting from training. Another well-documented, less complex yet multifunctional task is the 2-choice reaction time (2-CRT) task, which has been used to assess deficits in sustained attention (McGaughy and Sarter, 1995), motor readiness (Brown and Robbins, 1991), and response inhibition (Phillips and Brown, 1999; King et al., 2016), as well as overall attention deficits (Hausknecht et al., 2005). In the present study, a modified 2-CRT task was applied, which involved a short training process and thus could demonstrate possible attention deficits in young rats after PE.

Using an established second-trimester equivalent binge drinking PE model in our laboratory (Choong and Shen, 2004; Hausknecht et al., 2015; Wang et al., 2019), the present study aimed to efficiently demonstrate PE-induced attention deficits in both sexes of rats, which could pave a way for investigations of neural mechanisms underlying those deficits and explorations of effective intervention strategies in the future.

## Materials and Methods

### Animal Breeding and the PE Paradigm

Rats were bred in house, to eliminate prenatal stress caused by transportation. The breeding procedure has been described in detail before (Wang et al., 2019). Briefly, male and virgin female Sprague–Dawley rats (Envigo, Indianapolis, IN, United States) were housed together in breeding cages, food and water *ad lib*. The holding room was maintained with a 12 h/12 h light/dark cycle. Rat droppings were monitored on a daily basis until copulatory vaginal plugs were found (on gestational day/GD 0). Pregnant dams were then randomly assigned to the control or PE group and singly housed in standard plastic cages.

During GDs 8–20, pregnant dams were treated via intragastric gavage twice (5–6 h apart) every weekday during the light phase of the light/dark cycle, with 3 g/kg ethanol (15% w/v) or vehicle (22.5% w/v sucrose water, isocaloric to ethanol) per treatment. A single daily treatment with 4 g/kg solutions was given on weekends. The PE treatment is comparable to heavy prenatal alcohol exposure in humans (Eckardt et al., 1998; Shen et al., 1999). To make daily nutrient intake equivalent, controls were pair-fed with PE rats on GDs 8–20. All the rats were fed *ad lib* during other periods of time. In addition, dams received vitamin B injections (8 mg/kg; i.m.; twice a week) to prevent thiamine deficiency induced by ethanol exposure or the pair-feeding procedure (Roecklein et al., 1985; Ba et al., 1996). The advantage of gastric gavage is to control ethanol dosing precisely. Our previous data have shown that stress caused by gavage is minimal and well controlled (Hausknecht et al., 2015).

To minimize the potential impacts of PE dams’ alcohol withdrawal on maternal behavior, a cross-fostering procedure was performed, along with culling, on postnatal day (PD) 1. Each litter was randomly culled to 10 pups with equal numbers of males and females. Offspring of PE dams were transferred to foster dams who received no ethanol treatment and gave birth 2 days earlier than their PE counterparts. Control litters were cross-fostered pairwise among themselves. Weaning was conducted on PD 21. After weaning, same-sex rats were housed in pairs in standard plastic cages. Ninty-four rats eventually underwent the 2-CRT test (24 control males from 9 litters, 24 PE males/8 litters, 22 control females/8 litters, and 24 PE females/8 litters; 2.85 rats/litter on average). All the procedures conformed to the guidelines of the National Institutes of Health regarding laboratory animal care and use. The protocol was approved by the Institutional Animal Care and Use Committee of University at Buffalo.

### Apparatus

**FIGURE 2:**
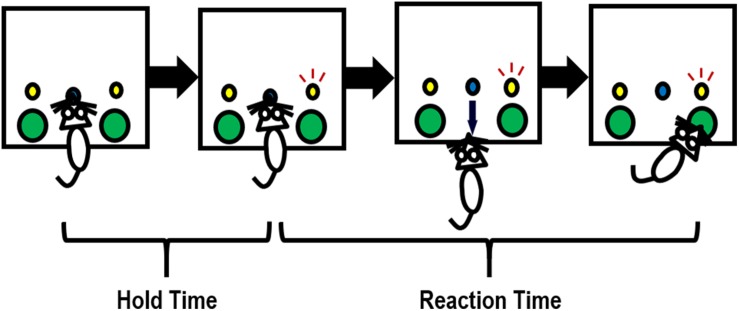
Illustration of how a rat completes one trial in the 2-choice reaction time task. Briefly, a snout poke into the center hole initiates a trial. The rat needs to hold its snout in the center hole until either the left or right stimulus light (randomly) turns on (e.g., the right stimulus light turns on in this figure). The holding period is referred to as *hold time*. Upon the illumination of the stimulus light, the rat needs to enter the water dispenser (a large hole) under the stimulus light to obtain a drop of water within a certain period of time (i.e., the allowed maximal *reaction time*). An incorrect response (entering a water dispenser not associated with the illuminating stimulus light) will terminate the trial; an extremely slow response (occurring when reaction time > the allowed maximal reaction time) will be considered an omission, with no water reward delivered.

Sixteen locally constructed operant chambers were used, of which detailed descriptions were provided previously (Richards et al., 1997; Hausknecht et al., 2005). Each chamber was located inside of a sound-and-light attenuating box (Rubbermaid, Atlanta, GA, United States), with a wall-mounted fan that provided ventilation and masking noise. The chambers had stainless steel grid floors, Plexiglas ceilings and front and back walls, and aluminum sides. In the test panel on the left side wall, there were two water dispensers located on both sides of a centrally located snout-poke hole. Above each of the two water dispensers was a stimulus light, as illustrated in Figure 2. Snout pokes into the center holes and head entries into the water dispensers were monitored with infrared detectors. Drops of water (0.03 ml/drop), as reinforcers, were delivered into the left and right dispensers by syringe pumps (PHM-100; MED Associates, Fairfax, VT, United States). All the chambers were connected to a computer with the MED Associates interface. The MED PC^®^ language was used to program the experimental contingencies.

### Procedure

A modified 2-CRT task was employed. Briefly, 6-week-old control and PE rats were water-restricted (water available for 0.5 h/day) so that drops of water served as a strong reinforcer in the test. The rats underwent 16–19 daily training sessions and 3 testing sessions, all conducted during the dark phase of the light/dark cycle. Each session lasted for 30 min. A trial was initiated by the rat inserting its snout into the center hole. The rat had to hold the snout inside the center hole for a period of time until the stimulus light turned on. This period was referred to as *hold time* (Figure 2). The hold time was cumulative. In other words, a rat could still meet the hold time requirement even if the hold was paused when the snout was pulled out before the hold time was up. Upon the onset of illumination of the stimulus light, the rat needed to rapidly withdraw from the center hole and enter into the water dispenser beneath the illuminating stimulus light to obtain a water reward. The time that elapsed during this process was defined as *reaction time* (RT, in Figure 2). An incorrect response (entering the water dispenser not associated with the illuminating stimulus light) terminated the trial immediately, with no water delivery. An extremely slow response (i.e., when RT > maximal trial duration, which was 2 s in testing sessions) was considered an omission, leading to no water reward as well.

In a regular trial, referred to as a *choice trial*, the stimulus light randomly illuminated either on the left or on the right side at the end of hold time. Besides choice trials, *forced trials* were also programed in to help rats avoid spontaneous alteration (Montgomery, 1951) and respond to stimuli correctly. Specifically, if the rat chose a wrong water dispenser (the one not associated with the illuminating stimulus light), a forced trial would occur afterward, in which the stimulus light above the previous correct water dispenser would turn on. Forced trials repeated until the rat eventually chose the right water dispenser. Rats were reinforced for correct timely responses in both choice trials and forced trials. In this paper, only the choice trials were analyzed. As such, a *trial* refers to a *choice trial* in the text below.

The maximal trial duration was defined as the maximal RT allowed in a trial, which was much greater than a typical RT. In order to selectively reinforce rapid responses, variable criterion RTs for reinforcement were introduced (Hausknecht et al., 2005). In each trial, if the actual RT > the criterion RT for that trial, no water reward was offered. The criterion RT was adjusted for every rat individually based on the following rules. If two correct responses were made in a row under the current criterion RT, the criterion RT for the next trial would decrease. If one incorrect or slow response (without reinforcement) was made, the criterion RT for the next trial would increase. The rules, therefore, could accommodate both fast- and slow-responders. The schedule of decrement/increment (in seconds) was 27.00, 10.00, 5.00, 2.50, 1.00, 0.89, 0.79, 0.71, 0.63, 0.56, 0.50, 0.45, 0.40, 0.35, 0.32, 0.28, 0.25, 0.22, 0.20, 0.18, 0.16, 0.14, 0.13, 0.12, 0.11, and 0.01. At the beginning of a session, the criterion RT was set at 0.71 s. Under the adjustments of criterion RTs, rats obtained reinforcements from approximately 75% of the correct responses (i.e., when the correct water dispensers were chosen), whether they responded fast or slow.

The rats’ behavior was shaped step by step. Specifically, to induce the initial poking behavior, in the first two sessions, drops of water were also available in the center hole contingent upon snout pokes. Within each of the first eight sessions, the stimulus light illuminated on the same side (left or right), to help rats establish the association between poking and availability of water rewards. From Session 9 on, the stimulus light turned on randomly on the left or the right side. Furthermore, the hold time increased gradually and transitioned from a fixed length to variable lengths within a session as training progressed. In the final three testing sessions, there were 20 different lengths of hold time, ranging from 0.08 to 12.6 s, with the mean hold time = 6 s (lengths of hold time in seconds: 0.0798, 0.246, 0.4212, 0.6066, 0.8034, 1.0134, 1.2414, 1.4814, 1.7466, 2.031, 2.3466, 2.697, 3.0918, 3.5436, 4.071, 4.7052, 5.514, 6.5712, 8.22, and 12.5868). In addition, the maximal duration of illumination of the stimulus light in a trial decreased from 3600 to 3 s and then to 1 s; the maximal trial duration (i.e., the maximal RT allowed) decreased from 3600 to 3 s and then to 2 s, as training progressed.

### Dependent Variables

To evaluate rats’ overall performance in the 2-CRT test and assess if PE caused any deficits in operant learning, the number of trials completed per session was tallied. To measure rats’ responding speed so as to assess if PE led to any motor deficits, the mean RT was compared between groups.

Premature responses were used as the major indicator of action impulsivity. Two types of premature responses were assessed: (1) a completed premature response, referred to as a false alarm, which was an entry into a water dispenser before the onset of illumination of any stimulus light, and (2) an incomplete premature response, or a premature initiation, which referred to pulling the snout out of the center hole and then quickly inserting it back, before the stimulus light turned on. False alarms/trial and premature initiations/trial were compared between groups.

**FIGURE 3:**
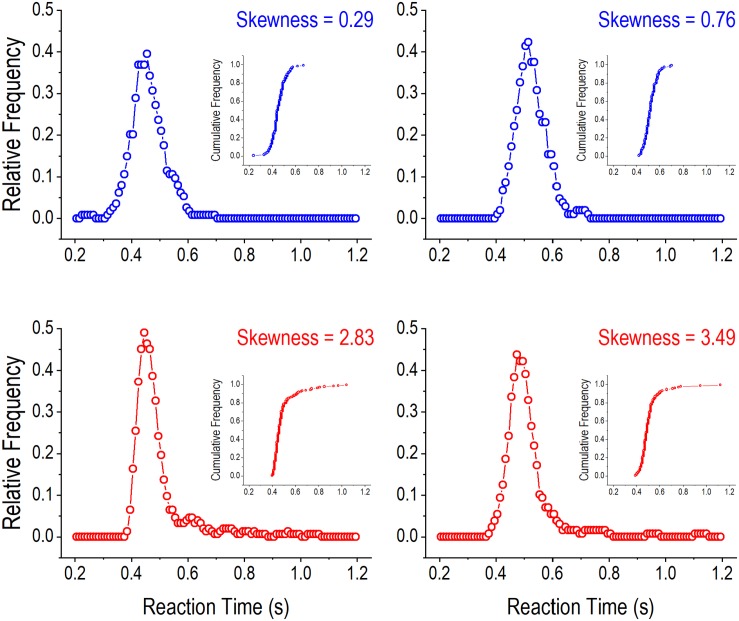
Illustration of reaction time (RT) distributions in the 2-choice reaction time task. The four panels show relative frequency distributions of RT in four rats when the hold time was greater than 4 s. In the **upper panels** (with blue curves), the smaller skewnesses of RT distributions are associated with shorter tails on the right side of the distribution curves. In contrast, in the **lower panels** (with red curves), the greater skewnesses of RT distributions are associated with longer tails on the right side of the distribution curves. One dot in an RT distribution curve represents the relative running frequency for a 50-ms bin (e.g., 200–250, 210–260, and 220–270 ms). The inserts are cumulative frequency distribution curves of RT in the four rats.

Lapses of attention lead to wrong or slow responses. The former were quantified by incorrect responses (in % of trials). Extremely slow responses led to omissions (measured as % of trials), which were not reinforced. Reinforced responses may also be relatively slow. Frequently occurring long RTs can positively skew the RT distribution, indicating lapses of attention, which has been observed in individuals with ADHD (Leth-Steensen et al., 2000). A positively skewed distribution is asymmetric, with a long tail on the right side of the distribution curve. Skewness of a distribution can be measured in different ways (Doane and Seward, 2011). In the present study, we used the *adjusted Fisher–Pearson standardized moment coefficient*, as computed by the following formula (Arnold and Groeneveld, 1995):

$$\text{Skewness}=n\,\frac{\sum {\left(X_{i}-{\text{Mean}}_{i}\right)}^{3}}{\left(n-1\right)\,\left(n-2\right)\times \sigma^{3}}$$

where σ was the standard deviation, and *n* was the number of trials. Figure 3 illustrates RT distribution curves with smaller (upper panels) and greater (lower panels) skewnesses, respectively. Greater skewnesses correspond to RT distribution curves with longer tails indicating the occurrences of long RTs. The distribution curves were constructed by grouping RTs into 50 ms bins (0–50 ms, 10–60 ms, 20–70 ms, etc.) and computing running relative frequencies for the bins (Hausknecht et al., 2005). The skewness statistic computed in this way is readily available in popular software programs, such as Excel.

### Data Analyses

All the analyses were based on the three testing sessions. A rat would be removed from data analyses if the average number of trials/session was <75 for males or <60 for females. The criterion was lower for females because female rats had lower bodyweights than males (by 20–35%) and thus they consumed less water. No male rats were excluded based on the criterion, whereas five control and four PE female rats were excluded.

For number of premature initiations/trial, number of false alarms/trial, and skewness of RT distribution, data were aggregated as two categories: when hold time was <4 s and >4 s, in order to separate easy from more difficult trials. As shown in the section “Results,” PE-induced deficits in male rats were only observed when the trials were more difficult. For number of premature initiations/trial and skewness of RT distribution, outliers (2.4% of all the data points) were detected using the Tukey fences (Zhou et al., 2006) and then winsorized (Winer, 1972) by being brought up/down to the next lowest/highest values in the same groups. This method of outlier detection and treatment has been used previously (Wang et al., 2018a, 2019).

To compare birth outcomes in pup number/litter, a 2-way analysis of variance (ANOVA, prenatal treatment: control vs. PE; sex: male vs. female) was utilized in statistical analysis. For all the other dependent variables, 2-way ANOVA with litter as a nested factor was applied. The nested ANOVA has been used previously (Wang et al., 2018a, b, 2019), as an effective way of controlling for potential litter effects, which was important because high correlations between littermates in a variety of parameters have been observed in studies involving prenatal treatments (Lazic and Essioux, 2013). Pairwise comparisons after 2-way ANOVA were performed using planned comparisons. Statistical programs SAS 9.4 (SAS Institute Inc., Cary, NC, United States) and Statistica 7 (Tibco Software Inc., Palo Alto, CA, United States) were employed for data processing and analysis. The significance level was set at α = 0.05. Data are presented as Mean ± SEM in the text and figures unless specified otherwise.

## Results

### Prenatal Ethanol Exposure Led to Slightly Lower Birthweights

Seventeen control and 16 PE dams gave birth to 215 and 222 pups, respectively (Table 1). PE had no impact on number of pups/litter (2-way ANOVA), but caused a small (3.09%) decrease in pup weight on PD 1 (2-way ANOVA with litter as a nested variable; main effect of prenatal treatment: *F*_(1_,_402)_ = 13.36, *p* < 0.001). Pup weights were also lower in females than in males (main effect of sex: *F*_(1_,_402)_ = 38.68, *p* < 0.001). Furthermore, litter had a significant effect on pup weights (*F*_(31_,_402)_ = 19.57, *p* < 0.001). No differences in bodyweight between control and PE rats of the same sex were observed at weaning (on PD 21) or in young adulthood (at the age of 8 weeks, data not shown). These results suggested that our PE paradigm did not lead to major teratogenic effects.

**TABLE 1:** Birth outcome after prenatal ethanol exposure.

	Control: 17 litters (Mean ± SEM)	PE: 16 litters (Mean ± SEM)	*p*-value
Litter size	12.65 ± 0.64	13.88 ± 0.53	0.258
Number of male pups	5.88 ± 0.55	6.63 ± 0.54	0.332
Number of female pups	6.76 ± 0.46	7.25 ± 0.58	0.524
**Pup weight on postnatal day 1**			
Average weight (g)	6.64 ± 0.06	6.44 ± 0.04	<0.001
Average male weight (g)	6.89 ± 0.08	6.60 ± 0.06	<0.001
Average female weight (g)	6.43 ± 0.07	6.29 ± 0.06	0.098

*Data are expressed as mean ± SEM. PE, prenatal ethanol exposure. *p*-values are based on planned comparisons after ANOVA.*

### Prenatal Ethanol Exposure Did Not Lead to Deficits in Operant Learning or Motor Behavior

**FIGURE 4:**
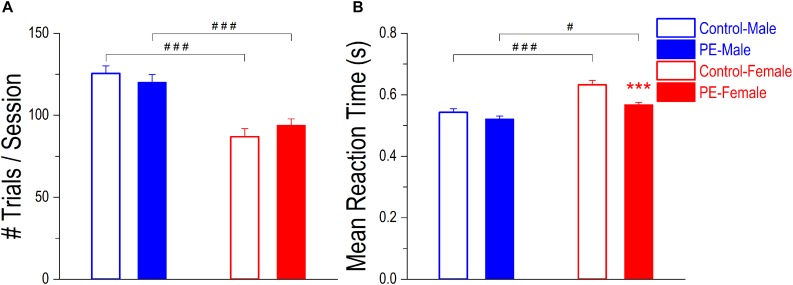
Prenatal ethanol exposure (PE) did not lead to operant learning or motor deficits. **(A)** Operant learning was not impacted by PE. After training, no differences were observed in number of trials completed per session between control and PE rats of the same sex. In addition, male rats completed more trials/session than their female counterparts with or without PE. **(B)** Prenatal ethanol exposure did not cause observable motor deficits. There was no difference in mean reaction time between control and PE male rats. In females, PE rats had even shorter mean reaction time than controls. In addition, male rats responded more rapidly than females with or without PE. Data are presented as Mean ± SEM. ***p < 0.001, control vs. PE in females. ^#^*p* < 0.05; ^###^*p* < 0.001, male vs. female with the same prenatal treatment.

Operant learning was not impacted by PE, based on rats’ overall performance in the 2-CRT task. Specifically, there was no difference in number of trials/session between control and PE rats in either sex (Figure 4A). Although a 2-way ANOVA with litter as a nested variable showed an interaction effect between prenatal treatment and sex (*F*_(1_,_61)_ = 4.54, *p* < 0.05), planned comparisons after ANOVA revealed no group difference between control and PE rats in either sex. Male rats completed more trials/session than females, probably because males had greater bodyweights and thus were motivated to obtain more water (number of trials/session in controls: male: 125.53 ± 4.62, female: 86.91 ± 4.97, *p* < 0.001, planned comparison; number of trials/session in PE rats: male: 120.10 ± 4.84, female: 93.78 ± 4.04, *p* < 0.001). Moreover, a litter effect was observed (*F*_(29_,_61)_ = 1.75, *p* < 0.05).

Prenatal ethanol exposure led to no observable motor deficits, in that PE did not lower the responding speed in the 2-CRT task (Figure 4B). Instead, PE female rats responded more rapidly (i.e., with shorter mean RT) than control females. Such a difference was not observed in males. A 2-way ANOVA with litter as a nested variable exhibited a main effect of prenatal treatment on mean RT (*F*_(1_,_61)_ = 19.04, *p* < 0.001). Planned comparisons after ANOVA revealed a significant difference in mean RT between control and PE females (control: 0.63 ± 0.014 s, PE: 0.57 ± 0.007 s; *p* < 0.001) and no difference between control and PE males (control: 0.54 ± 0.012 s, PE: 0.52 ± 0.010 s). Additionally, males responded more rapidly (i.e., with shorter mean RT) than females in both control and PE rats (main effect of sex: *F*_(1_,_61)_ = 31.72, *p* < 0.001; planned comparisons between control male and female rats: *p* < 0.001, and between PE male and female rats: *p* < 0.05).

### Prenatal Ethanol Exposure Increased Action Impulsivity Shown by Augmented Premature Responses

**FIGURE 5:**
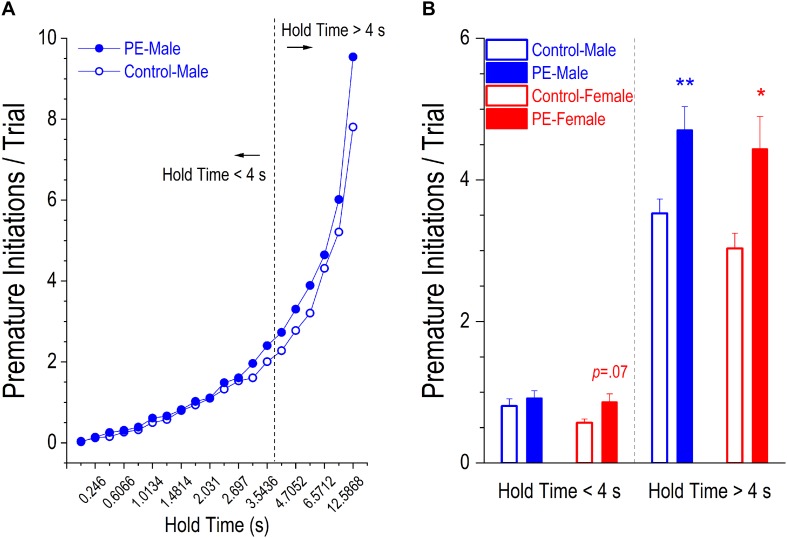
Prenatal ethanol exposure (PE) led to increased action impulsivity, shown by more premature initiations/trial in PE rats. A premature initiation refers to a snout withdrawal from and then quick insertion back into the center hole before the illumination of the stimulus light. **(A)** Depicts the average number of premature initiations/trial in control or PE male rats corresponding to each of the 20 different lengths of hold time. Visual inspection shows that the group difference became more marked as the hold time became longer. Therefore, two separate analyses were conducted for trials with the hold time < 4 s (i.e., when the trials were easier) and for those with the hold time > 4 s (i.e., when the trials were more difficult). **(B)** Rats with PE made more premature initiations/trial than controls in both sexes when the hold time was >4 s. In contrast, when the hold time was <4 s, there was a trend of a significant difference between control and PE rats in females (*p* = 0.07) but no group difference was observed in males. Data are presented as Mean ± SEM. *p < 0.05; **p < 0.01, control vs. PE of the same sex.

There were two types of premature responses displaying action impulsivity: incomplete, referred to as premature initiations, and completed, referred to as false alarms. PE led to an increase in number of premature initiations/trial. This effect was more prominent in more difficult trials with longer hold times (Figure 5A). As such, we analyzed group differences with shorter hold time (<4 s) and longer hold time (>4s) separately.

Rats with PE made significantly more premature initiations/trial than controls in both sexes when hold time was >4 s (Figure 5B). A 2-way ANOVA with litter as a nested variable revealed a main effect of prenatal treatment (*F*_(1_,_61)_ = 14.64, *p* < 0.001) with no main effect of sex. Planned comparisons after ANOVA showed a difference between control and PE males (control male: 3.53 ± 0.20, PE male: 4.70 ± 0.33; *p* < 0.01) and a difference between control and PE females (control female: 3.03 ± 0.21, PE female: 4.44 ± 0.46; *p* < 0.05).

In contrast, when hold time was <4 s, there was a trend of a significant difference in number of premature initiations/trial between control and PE females but no differences were observed between control and PE males (Figure 5B). A 2-way ANOVA with litter as a nested variable revealed a main effect of prenatal treatment (*F*_(1_,_61)_ = 5.14, *p* < 0.05) with no main effect of sex. Planned comparisons after ANOVA exhibited a trend of a difference between control and PE females (control female: 0.57 ± 0.05, PE female: 0.86 ± 0.11; *p* = 0.07), but no difference between control and PE males (control male: 0.81 ± 0.10, PE male = 0.92 ± 0.10).

**FIGURE 6:**
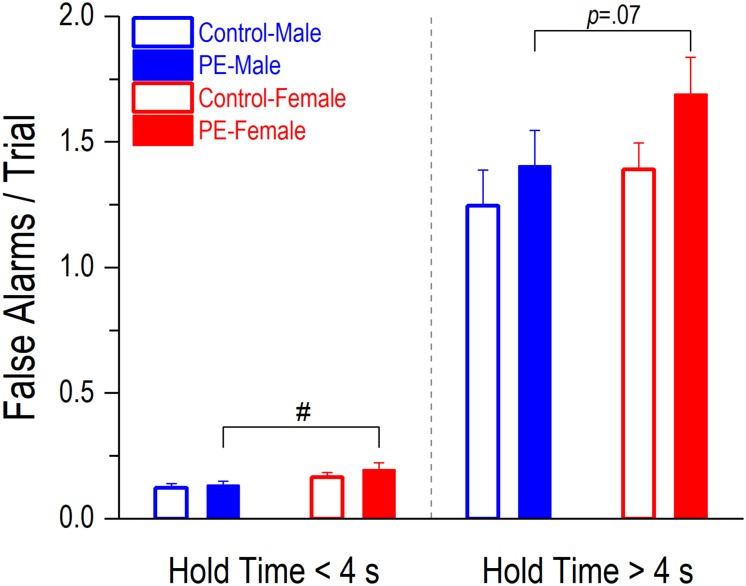
Prenatal ethanol exposure (PE) did not lead to an increase in false alarms/trial, regardless of the lengths of hold time. A false alarm refers to an entry into a water dispenser before the onset of illumination of any stimulus light, which is one of the indicators of action impulsivity. A sex difference was observed. Specifically, PE female rats made more false alarms/trial than PE male rats when the hold time was <4 s; a trend of such a difference was observed (*p* = 0.07) when the hold time was >4 s. Data are presented as Mean ± SEM. ^#^*p* < 0.05, PE male vs. female rats.

False alarms occurred less frequently than premature initiations (Figure 5B vs. Figure 6). PE did not lead to increased false alarms/trial regardless of the lengths of hole time but a sex effect was observed. Prenatally ethanol-exposed females made more false alarms than PE males (Figure 6). When hold time was >4 s, a 2-way ANOVA with litter as a nested variable showed a main effect of sex (*F*_(1_,_61)_ = 4.63, *p* < 0.05) and a litter effect (*F*_(29_,_61)_ = 2.54, *p* < 0.01). Planned comparisons following ANOVA revealed a trend of difference between the two sexes in PE rats (PE male: 1.41 ± 0.14, PE female: 1.69 ± 0.15; *p* = 0.07). No differences were observed between control males and females (control male: 1.25 ± 0.14, control female: 1.39 ± 0.11). Similarly, when hold time was <4 s, a 2-way ANOVA with litter as a nested variable produced a main effect of sex (*F*_(1_,_61)_ = 8.63, *p* < 0.01) and a litter effect (*F*_(29_,_61)_ = 2.30, *p* < 0.01). Planned comparisons showed a significant difference between the two sexes in PE rats (PE male: 0.13 ± 0.02, PE female: 0.20 ± 0.03; *p* < 0.05) while no differences were observed between control male and female rats (control male: 0.12 ± 0.02, control female: 0.17 ± 0.02).

### Prenatal Ethanol Exposure Led to Enhanced Lapses of Attention Demonstrated by More Skewed RT Distributions in PE Rats

**FIGURE 7:**
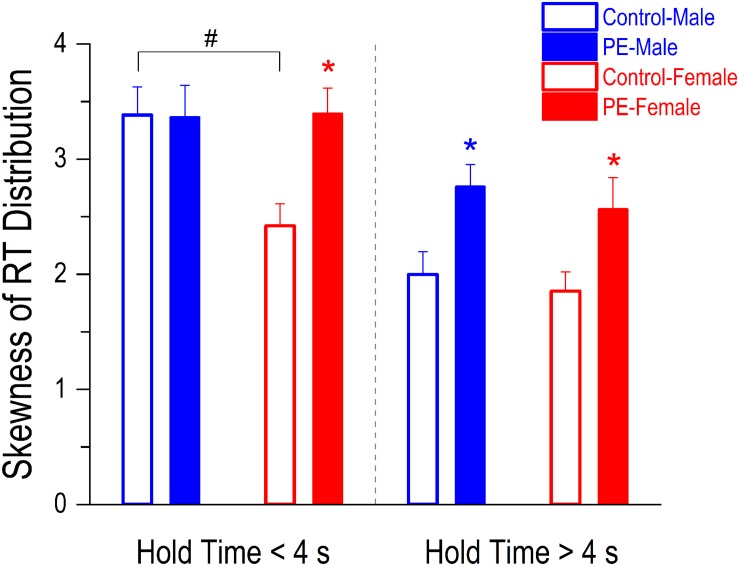
Prenatal ethanol exposure (PE) led to an increase in lapses of attention in both sexes when the trials were more difficult (i.e., when the hold time was >4 s). In contrast, the increase in lapses of attention was observed only in PE female rats but not in males when the trials were easier (i.e., when the hold time was <4 s). Lapses of attention were assessed by skewnesses of reaction time (RT) distributions (adjusted Fisher–Pearson standardized moment coefficient). The more the RT distribution curve is skewed positively (associated with excessive long RTs), the more lapses of attention occur in the test. In addition, a sex difference was observed in control rats. Specifically, the skewness of RT distribution was greater in control males than in control females when the hold time was <4 s. Data are presented as Mean ± SEM. *p < 0.05, control vs. PE of the same sex. ^#^*p* < 0.05, male vs. female in controls.

Individuals with attention deficits exhibit more positively skewed RT distributions due to the occurrence of excessive long RTs caused by lapses of attention. In more difficult trials (i.e., when hold time > 4 s), both PE male and female rats showed more lapses of attention than their control counterparts indicated by greater skewnesses of RT distributions in PE rats (Figure 7). A 2-way ANOVA with litter as a nested variable revealed a main effect of prenatal treatment (*F*_(1_,_61)_ = 9.82, *p* < 0.01) but no main effect of sex. Planned comparisons after ANOVA showed a difference between control and PE males (control male: 2.00 ± 0.20, PE male: 2.76 ± 0.19; *p* < 0.05) and a difference between control and PE females (control female: 1.85 ± 0.17, PE female: 2.56 ± 0.27; *p* < 0.05).

In less difficult trials (when hold time < 4 s), increased lapses of attention in PE rats were only observed in females but not in males perhaps because control male rats already had more lapses of attention than control females (Figure 7). A 2-way ANOVA with litter as a nested variable produced a main effect of sex (*F*_(1_,_61)_ = 3.62, *p* = 0.06). Planned comparisons after ANOVA revealed a difference between control and PE females (control female: 2.42 ± 0.19, PE female: 3.40 ± 0.22; *p* < 0.05) while no differences were observed between control and PE males (control male: 3.38 ± 0.24, PE male: 3.36 ± 0.28). For rats without PE, males showed more lapses of attention than females in less difficult trials (*p* < 0.05).

**FIGURE 8:**
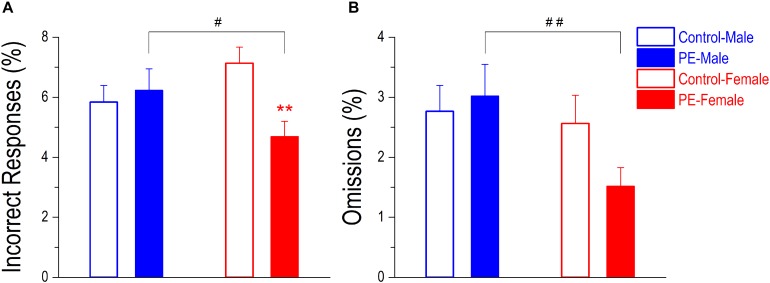
Prenatal ethanol exposure (PE) did not lead to more incorrect responses or omissions in the 2-choice reaction time task. These two types of errors both indicate lapses of attention. An incorrect response refers to an entry into a wrong water dispenser (which is not associated with the illuminating stimulus light) at the right time. An omission refers to an extremely slow response with reaction time > the maximal trial duration. These two types of errors occurred infrequently in well-trained rats, but a sex difference was observed. Specifically, PE caused no change in incorrect response (in % of trials, **A**) or omission (in % of trials, **B**) in males whereas PE led to reduced incorrect responses in females. Moreover, PE females made fewer incorrect responses and omissions than PE males. Data are presented as Mean ± SEM. **p < 0.01, control vs. PE in females. ^#^*p* < 0.05; ^##^*p* < 0.01, male vs. female in PE rats.

Lapses of attention can also be manifested as incorrect responses (poking into the wrong water dispenser) or omissions (when RT > maximal trial duration). Nevertheless, these errors occurred infrequently and were not major indicators of attention deficits in the 2-CRT task. PE did not cause an increase in percentage of incorrect responses in males. In contrast, PE decreased incorrect responses in females despite their rare occurrences (Figure 8A). A 2-way ANOVA with litter as a nested variable produced an interaction effect between prenatal treatment and sex (*F*_(1_,_61)_ = 7.83, *p* < 0.01). Planned comparisons after ANOVA showed a difference between control and PE females (control female: 7.13 ± 0.54%, PE female: 4.69 ± 0.52%; *p* < 0.01). There was no difference between control and PE males (control male: 5.84 ± 0.55%, PE male: 6.23 ± 0.72%). In addition, PE males made more incorrect responses than PE females (*p* < 0.05).

Prenatal ethanol exposure did not lead to an increased percentage of omissions, but PE males made more omissions than PE females (Figure 8B). A 2-way ANOVA with litter as a nested variable showed a main effect of sex (*F*_(1_,_61)_ = 4.68, *p* < 0.05). Planned comparisons after ANOVA revealed a difference between the two sexes in PE rats (PE male: 3.02 ± 0.53%, PE female: 1.52 ± 0.31%; *p* < 0.01). No differences were observed between the two sexes in control rats (control male: 2.76 ± 0.43%, control female: 2.56 ± 0.47%).

## Discussion

The major finding of the present study is that PE leads to increased action impulsivity and lapses of attention in both sexes, indicating that PE produces major impairments in the attentional process throughout the pre-cue and post-cue stages. The deficits in action impulsivity and lapses of attention correspond to hyperactivity/impulsivity and inattention observed in ADHD individuals, respectively, supporting that the PE paradigm we used serves as a valid rodent model to demonstrate PE-induced attention deficits.

Prenatal ethanol exposure leads to slightly lower birthweight but exerts no impact on litter size, or bodyweight at weaning or in young adulthood, indicating that our PE treatment modeling binge drinking does not induce major teratogenic effects, as consistently shown in our previous studies (Hausknecht et al., 2015; Wang et al., 2018b, 2019). In the 2-CRT task, no differences are observed in number of trials completed between control and PE rats of either sex, indicating that PE causes no deficits in operant learning, which is consistent with our finding from a previous study on drug self-administration in rats (Wang et al., 2019). Although they show no deficits in learning, PE rats exhibit attention impairments, which is in line with the clinical observation that many children with FASD are diagnosed with ADHD, even though they have normal IQs.

We also observe that PE male rats respond as rapidly as control males in the 2-CRT task, shown by their similar RTs. Interestingly, PE female rats respond even faster than control females. These observations suggest that PE leads to no motor deficits. As such, other group differences detected in the 2-CRT test should not be attributed to learning or motor issues that might be produced by PE.

One interesting observation is that across treatment groups, male rats completed more trials/session than females (Figure 4A), and males respond faster (with shorter reaction time) than females (Figure 4B). This is probably due to the greater bodyweights in males than in females (by 20–35%, regardless of the prenatal treatments), which leads to higher water volume requirement in males. Also, faster responding in males could result from their relative ease moving from one poke hole to another because of their larger physiques.

It appears that PE impacts female rats to a greater extent than males in the attentional process. For example, PE female rats exhibit more false alarms than PE male rats (Figure 6), which sex difference is not observed in controls. In addition, relative to control males, increased action impulsivity (in terms of premature initiations) and lapses of attention are exhibited in PE male rats only when the trials are more difficult (i.e., hold time > 4 s). In contrast, these deficits are observed in PE female rats even when the trials are relatively easy (i.e., hold time is <4 s). A possible reason for this effect is that control male rats show more deficits than control females when hold time is <4 s, including a trend of higher impulsivity (Figure 5B) and significantly greater lapses of attention (Figure 7). The existing sex differences in controls might lead to more limited PE effects in males. Our observations suggest that control males are more likely to exhibit attention deficits than control females, which is consistent with the gender differences found in clinical studies. In 6–18-year-old children, the ratio of males/females diagnosed with ADHD ranges from 3:1 to 16:1 in different countries (Nøvik et al., 2006). There are also sex differences in the nature of ADHD. Boys are more likely to exhibit hyperactive/impulsive symptoms while inattention symptoms are more often observed in girls (Newcorn et al., 2001; Biederman et al., 2002; Biederman and Faraone, 2004). Sex differences in attention deficits in children with FASD have not been thoroughly investigated. To our knowledge, there is only one study showing that males (68%) are more likely to be diagnosed with ADHD than females (29%), but no sex differences in behavioral measures of attention are observed (Herman et al., 2008). We believe the results of the present study could provide unique insights into sex differences in attention deficits in individuals with FASD.

The PE paradigm applied in the present study (6 g/kg/day via two gastric intubations, 15% w/v ethanol) corresponds to the second trimester-equivalent of heavy drinking in humans (Eckardt et al., 1998; Shen et al., 1999). The method of ethanol administration, gastric gavage, could precisely control alcohol volumes administered, and the dose we applied is commonly used in preclinical PE studies (Gil-Mohapel et al., 2010). Importantly, in the present study, we have implemented a few control procedures to minimize the impacts of potential confounding factors in order to ensure that observed behavioral effects are indeed due to exposure to ethanol. The procedures include: (1) pair-feeding the control dams with dams exposed to ethanol, so as to maintain nutritional equivalence between the two groups; (2) administering vitamin B to eliminate thiamine deficiency caused by ethanol intake, which can impact brain function; and (3) using dams with no ethanol treatment to foster the PE pups in order to avoid disrupted maternal behavior due to alcohol withdrawal. These procedures could enhance the isolation of the ethanol effect but they might sacrifice the external validity of the animal model to some extent. For example, it has been reported that a major factor associated with fetal alcohol syndrome (FAS), i.e., the most severe conditions caused by heavy prenatal alcohol exposure, is low socioeconomic status in Europe and the United States (Abel, 1995). This implies that mothers and the fetuses/infants might experience other adversities (e.g., undernutrition, increased stress, and abuse). As a result, the final outcomes of PE are not solely determined by the ethanol effect. In the future, it is important to properly model the interactions between PE and other adverse pre- and postnatal factors existing in humans to truly understand the deficits in FASD. In fact, new studies on such interaction effects have been emerging (Raineki et al., 2017; Lam et al., 2018).

The 2-CRT task utilized in the present study only requires a relatively short training process, compared with the traditional 2-CRT paradigm (Phillips and Brown, 1999; Hausknecht et al., 2005). Accordingly, the PE-induced attention deficits can be observed in young adulthood (≤10 weeks old), which makes the observations in the task better correspond to human conditions, since ADHD is a developmental disorder with early onset (American Psychiatric Association [APA], 2013). Nevertheless, PE-induced attention deficits could be persistent, because a previous study also shows enhanced impulsivity and lapses of attention in older PE male rats (Hausknecht et al., 2005). In addition, another advantage of having a shorter training process is, as mentioned earlier, to avoid introducing a variety of possible confounding factors associated with protracted training.

Among the two impulsivity measures, false alarms are completed premature responses (entering a water dispenser before any stimulus light turns on), which, compared with premature initiations (coming out of the center hole prematurely without entering any water dispenser), consume much more energy and are infrequently observed. Premature initiations, in contrast, occur more frequently. This observation is consistent with previous studies (Sabol et al., 2003). However, different from the 2-CRT task, in 5-choice or 3-choice serial reaction time tasks, only false alarms are observed because the animal is not required to hold the snout in a hole (Robbins, 2002; Bari et al., 2008). As such, the 2-CRT task has an advantage in better detecting premature responses caused by action impulsivity.

Skewness of RT distribution is an important dependent variable for assessing lapses of attention in the present study. It is computed using the adjusted Fisher–Pearson standardized moment coefficient. In several earlier studies including our own, skewness was computed based on deviation of the mode from the mean – DevMode (Sabol et al., 2003; Hausknecht et al., 2005). A commonly occurring problem with such an approach is that an RT distribution could have multiple modes, which makes it difficult to compute DevMode in certain cases. In addition, DevMode is essentially the difference between two centrality measures – mean and mode. As such, it does not necessarily reflect the tail size of a distribution curve, which is a critical factor determining the magnitude of skewness. The adjusted Fisher–Pearson standardized moment coefficient, in contrast, is a reliable measure of the tail size because it amplifies the impact of larger numbers in a distribution by having ∑(*X*_*i*_ − Mean_*i*_)^3^ in the numerator of the formula. Admittedly, a weakness of this measure is that the result can be sensitive to a few extreme numbers in a distribution. However, by carefully examining each distribution of RT, we have found that pronounced skewness is always caused by the occurrence of a continual string of relatively long RTs in the distribution (Figure 3) corresponding to enhanced lapses of attention. Additionally, the skewness measure is not based on curve fitting, and thus it is not constrained by *a priori* assumptions. Taken together, we believe that the adjusted Fisher–Pearson standardized moment coefficient is a more suitable method for analyzing skewness of reaction time and inferring lapses of attention.

Incorrect responses and omissions are two other types of errors indicating lapses of attention. They, however, are rarely observed in the 2-CRT task, possibly because the difficulty level of the task is relatively low. Accordingly, the results involving there two dependent variables should not be overinterpreted, as mentioned above. The incapability of relying on incorrect responses and omissions to infer the attentional process is a major disadvantage of the 2-CRT task in its current form when compared with the 5-choice or 3-choice serial reaction time task. In order to fully understand how PE impacts attention, it is important to assess these two types of errors displaying lapses of attention. In the future, modifications aiming to increase the difficulty of the task will be attempted, such as introducing distractors (house light or noise), shortening the duration of illumination of the stimulus light, and adding a third choice.

Attentional control is not an isolated cognitive function. Instead, it is often considered to be a component of “executive function,” which consists of working memory, inhibitory control, cognitive flexibility (set-shifting), planning, fluency, and other interdependent components (Barkley, 1997; Willcutt et al., 2005; Breckenridge, 2007). All of them work together to guide goal-directed behaviors (Kodituwakku et al., 2001a). Besides attention deficits, individuals with FASD also exhibit other cognitive deficits involving executive function, which may, in turn, negatively influence attentional control as well (Kingdon et al., 2016). Furthermore, it has been suggested that FASD show deficits in executive function related to altered emotional state (e.g., anxiety) (Rolls et al., 1994; Dias et al., 1996). This is demonstrated in tasks involving rewards or punishments when emotionally charged stimuli are applied (Kodituwakku et al., 2001a, b). These observations are not surprising, considering that PE leads to increased anxiety (Hellemans et al., 2008; Walthall et al., 2008; Hellemans et al., 2010). In addition, overlapping brain areas, such as the prefrontal cortex (PFC), the posterior parietal cortex, and multiple cortical and limbic structures (Ball et al., 2011; Yuan and Raz, 2014), control both emotional behaviors and executive function. Animal models are indispensable in delineating the networks that connect these brain areas and elucidating how they are impaired by PE. Among these cortical and limbic structures, the PFC plays a significant role in executive function by exerting its top-down control over other cortical and subcortical brain regions (Tomita et al., 1999; Koechlin et al., 2003; Koechlin and Summerfield, 2007). The medial PFC (mPFC) in rats, corresponding to the dorsolateral PFC in humans, is crucial for attentional control (Gill et al., 2000; Totah et al., 2013). Behavioral studies have shown that lesions to the mPFC lead to attentional impairments (Broersen and Uylings, 1999; Maddux and Holland, 2011; Kahn et al., 2012). The mPFC is composed of anterior cingulate cortex, prelimbic cortex (PL), and infralimbic cortex (Heidbreder and Groenewegen, 2003), among which the PL appears to be involved in all components of the attentional process (Totah et al., 2013; Sharpe and Killcross, 2018). Using a similar 2-CRT paradigm, Hardung et al. (2017) shows that optogenetically inhibiting pyramidal neurons in the PL increases premature responses in naïve rats. Accordingly, we speculate that neuronal dysfunction in the PL leads to attention deficits observed in PE rats. This possibility is being studied in our laboratory.

In the present study, we have demonstrated that PE indeed leads to attention deficits in rodents using a 2-CRT task. This model allows us to investigate the neural mechanisms underlying deficits in executive function caused by PE, which will facilitate the development of effective intervention approaches for FASD. In addition, it has been reported that prenatal exposure to stress (Rodriguez and Bohlin, 2005; Ronald et al., 2011) or substances such as cocaine (Linares et al., 2005; Morrow et al., 2009), heroin (Ornoy et al., 2001), caffeine (Bekkhus et al., 2010), and nicotine (Milberger et al., 1996; Ernst et al., 2001; Rodriguez and Bohlin, 2005), also leads to an increased risk of ADHD. As such, our 2-CRT task may be used to study attention deficits caused by other prenatal risk factors. Taken together, these investigations will help better understand the pathophysiology of ADHD.

## Data Availability Statement

The datasets generated for this study are available on request to the corresponding author.

## Ethics Statement

The animal study was reviewed and approved by the Institutional Animal Care and Use Committee of University at Buffalo.

## Author Contributions

RW, CM, AL, KH, and KI performed behavioral tests and completed data analyses. KH and R-YS conducted prenatal ethanol treatments. R-YS and JR directed the project. R-YS, JR, KH, RW, and SH-D participated in the experimental design. RW and R-YS wrote the manuscript.

## Conflict of Interest

The authors declare that the research was conducted in the absence of any commercial or financial relationships that could be construed as a potential conflict of interest.
